# Cartilage reconstruction using Chondrofiller in intra-articular distal radius fractures

**DOI:** 10.1186/s42836-025-00333-y

**Published:** 2025-10-06

**Authors:** Wolfram Demmer, Antonia Zörnpfenning, Kevin Brugetti, Sinan Mert, Elisabeth Haas-Lützenberger, Riccardo Giunta, Irene Mesas Aranda

**Affiliations:** Department of Hand, Plastic and Aesthetic Surgery, Ludwig-Maximilians-University Munich, 80336 Munich, Germany

**Keywords:** Intra-articular distal radius fracture, Cartilage reconstruction, Chondrofiller, Wrist arthroscopy, Post-traumatic osteoarthritis

## Abstract

**Introduction:**

Intra-articular distal radius fractures (DRF) frequently result in post-traumatic cartilage defects, which can lead to early osteoarthritis, particularly when residual step-offs or gaps ≥ 2 mm remain after fracture reduction. While cartilage repair techniques are well established in large joints, options for small joints like the wrist remain limited. Chondrofiller liquid, a cell-free collagen type I hydrogel, has demonstrated regenerative potential in larger joints; however, its application in the wrist has not yet been studied.

**Methods:**

In this prospective clinical study, 59 patients with intra-articular DRFs treated by arthroscopy-assisted open reduction and internal fixation (ORIF) were included. In 25 cases, residual chondral defects between 0.5 and ≤ 2 mm were filled with Chondrofiller. Technical feasibility, defect characteristics, and application parameters were recorded. A subgroup of 8 Chondrofiller-treated patients underwent follow-up arthroscopy and was compared to a matched control group (*n* = 7) that had received ORIF without Chondrofiller. Cartilage quality was assessed using Outerbridge and the International Cartilage Repair Society (ICRS) classifications.

**Results:**

Chondrofiller was applied arthroscopically under dry conditions using G20–21 cannulas. Only 0.2–0.3 mL of the 1 mL preparation was required in most cases. At follow-up arthroscopy, patients treated with Chondrofiller showed significantly better cartilage quality (median Outerbridge 1.5 vs. 3, *P* = 0.006; ICRS 1 vs. 3, *P* = 0.002). Fibrous tissue formation (FTF) occurred only in overfilled defects, while flush applications were free of FTF. No significant differences were found in consolidation, complications, or associated injuries between groups.

**Conclusion:**

Chondrofiller is technically feasible and safe for use in the wrist. When applied precisely and flush with the cartilage surface, it may improve cartilage quality after intra-articular DRF. Further long-term clinical and radiological studies are needed to assess its effectiveness in preventing post-traumatic osteoarthritis and to evaluate cost-effectiveness compared to standard treatment.

## Introduction

The distal radius fracture (DRF) is consistently reported as one of the most common fractures in humans [1–4]. The incidence as well as the surgical treatment of DRF has been increasing over the past decades [5–7]. Distal radius fractures are classified according to the AO/OTA classification into extra-articular fractures of the radial metaphysis (23-A, 1–3), fractures with partial articular involvement (23-B, 1–3), and intra-articular fractures (23-C, 1–3) [8]. Intra-articular DRFs with partial or complete destruction of the radial articular surface (23-B and 23-C fractures) are reported in one to two-thirds of all DRFs [2, 9]. Due to instability or significant displacement with step formation, open reduction and volar plate osteosynthesis is considered the gold standard for the treatment of intra-articular DRF [10–13]. The restoration of articular congruity in displaced articular fragments is one of the primary goals in the Management of intra-articular DRF. The considerable damage to the articular surface associated with intra-articular distal radius fractures can lead to post-traumatic radiocarpal osteoarthritis, resulting in pain, restricted range of motion, and loss of strength in the wrist and hand. The prevalence of radiocarpal osteoarthritis following distal radius fractures in non-osteoporotic patients is reported to be as high as 35–40% [14, 15]. In line with Matta’s classification, an anatomic reduction of the articular surface is aimed for, with a step-off or gap of up to 1 mm generally being tolerated, whereas step-offs or gaps of ≥ 2 mm are significantly associated with a higher incidence of post-traumatic osteoarthritis [16–21].

Arthroscopy-assisted treatment of intra-articular distal radius fractures not only allows for the identification and management of ligamentous or cartilaginous soft tissue injuries but also offers the advantage of directly assessing and, if necessary, improving the fracture reduction [22–24]. In comparison to fracture reduction under fluoroscopy alone, arthroscopy-assisted reduction enables improved congruency of the displaced fragments of the distal radius joint surface [25–27]. In particular, persistent gaps in the articular joint surface can be identified more accurately with arthroscopy than with fluoroscopy alone. [26, 28].

Post-traumatic gaps in the joint surface with exposed subchondral bone represent Grade 4 cartilage defects according to both the Outerbridge and International Cartilage Repair Society (ICRS) classifications [29, 30]. Even focal Grade 4 cartilage defects can result in significant damage to the overlying cartilage and corresponding subchondral bone, ultimately leading to the development of osteoarthritis [31, 32].

While the subchondral bone can typically bridge the gap and heal *ad integrum*, cartilage defects are filled by fibroblasts rather than chondrocytes, resulting in the formation of a chondral scar. [33].

Collagen in various forms is currently under investigation or is already being used for osteochondral therapy [34]. Chondrofiller liquid (Meidrix biomedicals GmbH, Esslingen, Germany) is a type I collagen hydrogel that is already clinically used for arthroscopic therapy of chondral defects [35]. In animal studies, it has been demonstrated that this cell-free hydrogel triggers migration and proliferation of chondrocytes, ultimately resulting in the formation of high-quality regenerative tissue [36, 37]. Studies on the use of Chondrofiller liquid for osteochondral lesions of the hip and knee have also reported promising results [37–40].

There are currently no studies on the use of Chondrofiller liquid in the wrist, particularly for the treatment of residual chondral lesions following intra-articular distal radius fractures. This study aims to examine the technical feasibility in the comparatively small joint cavity of the wrist. Indication parameters for the rational use of the product are to be provided, and technical experiences gained during these investigations are to be shared. As a preliminary result, the outcomes of the first follow-up arthroscopies, conducted more than 6 months after fracture treatment, are presented and compared to intra-articular fractures not treated with Chondrofiller liquid.

## Materials and methods

In this prospective clinical trial, patients with an intra-articular distal radius fracture treated with an arthroscopy-assisted procedure were examined between February 2023 and January 2025. Inclusion criteria were a medical indication for surgical treatment of an intra-articular distal radius fracture (DRF) by open reduction and internal fixation (ORIF) using a volar plate, followed by wrist arthroscopy to evaluate and, if necessary, adjust the reduction outcome, as well as a patient age over 18 years. General exclusion criteria were a DRF without an indication for surgical and arthroscopically assisted treatment, patient refusal of surgical treatment, treatment of the DRF by any method other than volar plate osteosynthesis, and patient age under 18 years. Ethical approval for this study was granted by the Ethics Committee of the Medical Faculty of Ludwig Maximilian University of Munich, Germany (No. 24–0270, Date: 04/09/2024). Informed consent was obtained from all participants.

Step-offs in the joint surface were tolerated up to 1 mm, and gaps in the joint surface were tolerated up to 2 mm. In cases where gaps between 0.5 and 2 mm were present, these were minimally invasively filled with Chondrofiller liquid. Photo and video documentation were performed. In some patients, a re-arthroscopy was conducted more than 6 months after fracture treatment. The re-arthroscopy was performed for medical indications only, in cases of persistent wrist complaints. In these instances, the previously fractured joint surface and its cartilage cover were evaluated according to the Outerbridge and ICRS criteria (Table 1).

**Table 1:** The Outerbridge Classification and ICRS Classification of intra-articular cartilage damage [29, 41]

Grade	Outerbridge Classification	ICRS Classification**
Grade 0	Normal cartilage	Normal cartilage
Grade 1	Softening and swelling of the cartilage	fissures and cracks
Grade 2	Fragmentation and fissuring < 1.5 cm in diameter*	Lesions extending down < 50% of cartilage depth
Grade 3	Fragmentation and fissuring > 1.5 cm in diameter*	Lesions > 50% of cartilage depth down to the calcified layer, but not through the subchondral bone
Grade 4	Erosion down to the subchondral bone (exposed bone)	Lesions extending through subchondral bone (full-thickness defect)

^*^Original description converted to metric, **Simplified as published by LaPrade et al.

**Fig. 1:**
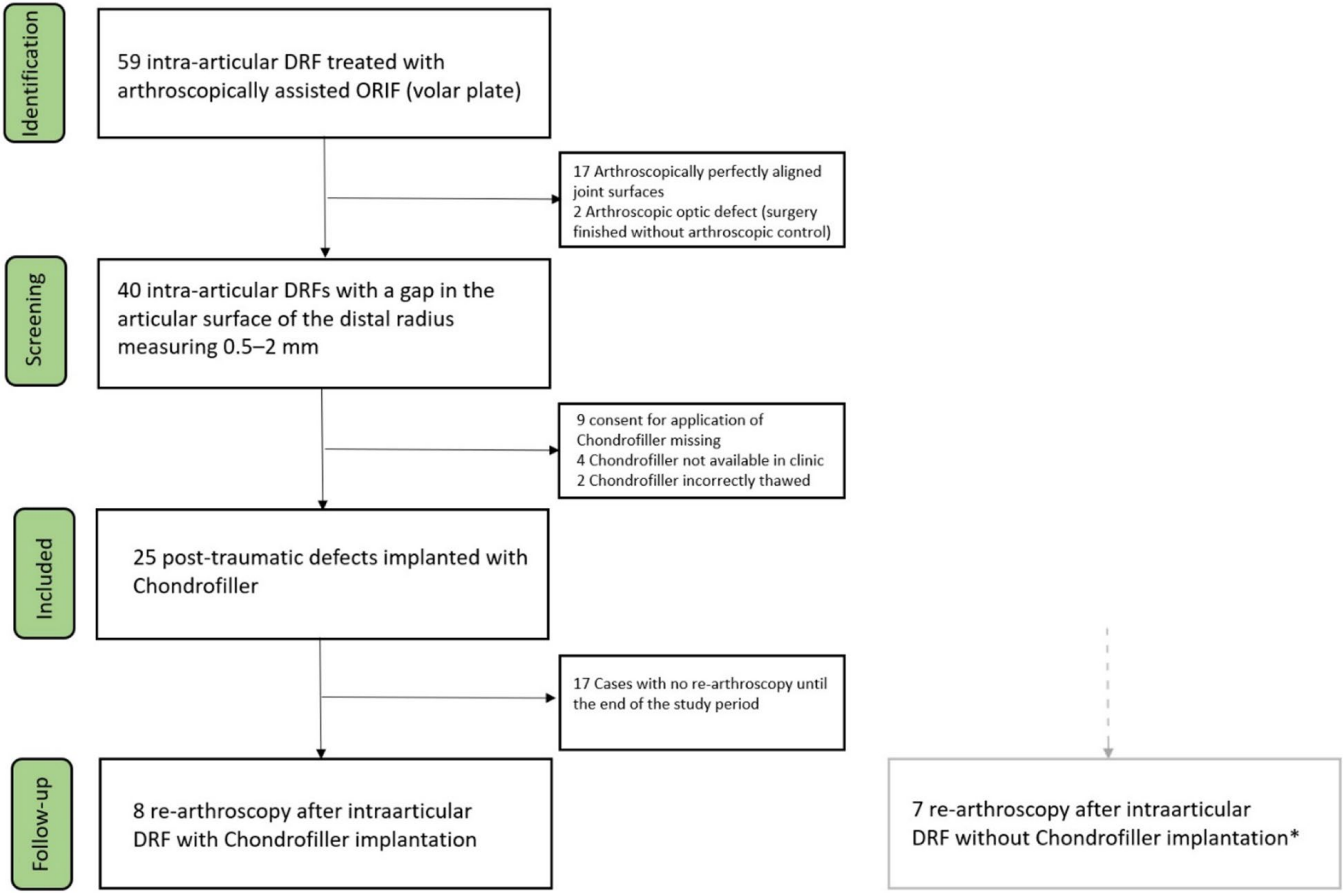
Study Flow Chart. *All re-arthroscopies of DRF without the use of Chondrofiller within the study period were included. Primary treatment did not necessarily take place during the study period or in our clinic

The follow-up results of Chondrofiller-treated intra-articular fractures were compared with matched intra-articular DRF cases that were initially not treated with Chondrofiller liquid for various reasons and subsequently underwent re-arthroscopy (Fig. 1).

### Surgical technique

Osteosynthesis was performed using standard ORIF and a volar locking plate (Medartis AG, Basel, Switzerland), with release of the brachioradialis from the radial styloid, if necessary, as commonly described [42–44]. The primary reduction and fixation were performed under fluoroscopic control. After reduction and stabilization, the injured arm was suspended in an arthroscopy holder. Subsequently, diagnostic arthroscopy was performed using a 2.7 mm 30° optic (Karl Storz SE & Co. KG, Tuttlingen, Germany) through 3/4 and 6R standard portals. Routine inspection of the ligamentous and cartilaginous components of the wrist joint was conducted. The radial joint surface was examined for step-offs and gaps. Step-offs of up to 1 mm and gaps ≤ 2 mm were tolerated. If larger step-offs or gaps in the joint surface were detected, the reduction was redone under arthroscopic visual control using K-wires and repositioning of the locking screws. Gaps ranging from 0.5 mm to ≤ 2 mm were routinely filled with Chondrofiller liquid. For this purpose, the arthroscopy was performed dry, and the curing of the hydrogel was observed under direct vision. Postoperatively, immobilization was Maintained for 2 weeks before starting non-weight-bearing physiotherapeutic exercises while still in the splint.

### Chondrofiller liquid storage and application

Chondrofiller liquid is a type I collagen hydrogel. It is supplied as a dual-chamber syringe containing separate compartments for collagen type I and the neutralization solution. Through the uniform mixing of the chamber components during injection, the hydrogel cures at body temperature, turning opaque. Chondrofiller was stored as recommended by the manufacturer and thawed timely manner before use [45]. The smallest package sizes (1 mL) were used. Application was performed into the fracture gap under direct vision using a 20 G or 21 G cannula (Becton Dickinson GmbH, Heidelberg, Germany).

All findings were documented arthroscopically with photos and video. To calibrate size ratios in the analysis of the image Material, the 2 mm-long head of the probe was used as a reference (Karl Storz SE & Co. KG, Tuttlingen, Germany). The measurement of defects was then performed using ImageJ (Version 1.54p, Open Source). If intraoperative images were not assessable, gaps were evaluated in post-operative X-rays (p.a. and lateral). The assessment of follow-up cartilage quality was conducted by board-certified hand surgeons using the Outerbridge and ICRS criteria (Grades I-IV).

### Statistical analysis

Continuous variables are reported as means with standard deviations or as medians with minimum and maximum values, depending on data distribution. Categorical variables are presented as absolute and relative frequencies. Normality was assessed using the Shapiro–Wilk test; homogeneity of variances with Levene’s test. A *P*-value ≤ 0.05 was considered statistically significant. Analyses were performed using IBM SPSS Statistics, Version 28.0 (IBM Corp., Armonk, NY, USA).

Depending on data type and distribution, appropriate tests were applied: independent samples t-test or Mann–Whitney U test for group comparisons, binomial and chi-square tests for categorical data. Cross-tabulations were used to examine associations between categorical variables, including Fisher’s exact test where appropriate. Two-way ANOVA was applied to assess interaction effects, and binary logistic regression to evaluate predictors of dichotomous outcomes.

Graphs were created using IBM SPSS Statistics, Version 28.0 (IBM Corp., Armonk, NY, USA). Illustrations were created by the author with the assistance of ChatGPT-4o (OpenAI, San Francisco, CA, USA).

## Results

### Technical results

Chondrofiller liquid can be stored at − 35 to − 15 °C for 24 months after production. Before use, it should be thawed in a refrigerator at 2 to 8 °C for over 24 h. Once warmed above 8 °C, it must be used within 24 h. Refreezing is not possible [45]. Since the indication for the use of Chondrofiller in our clinic is more than once per month, one unit of Chondrofiller is continuously stored at 2 to 8 °C. Packaging sizes of 1.0 mL, 1.5 mL, and 2.3 mL are available [45]. In this study, 1.0 mL was used for each procedure. After discarding the first drop, as advised by the manufacturer, and carefully filling the defect, approximately three-quarters of the Chondrofiller liquid remained in the syringe.

**Fig. 2:**
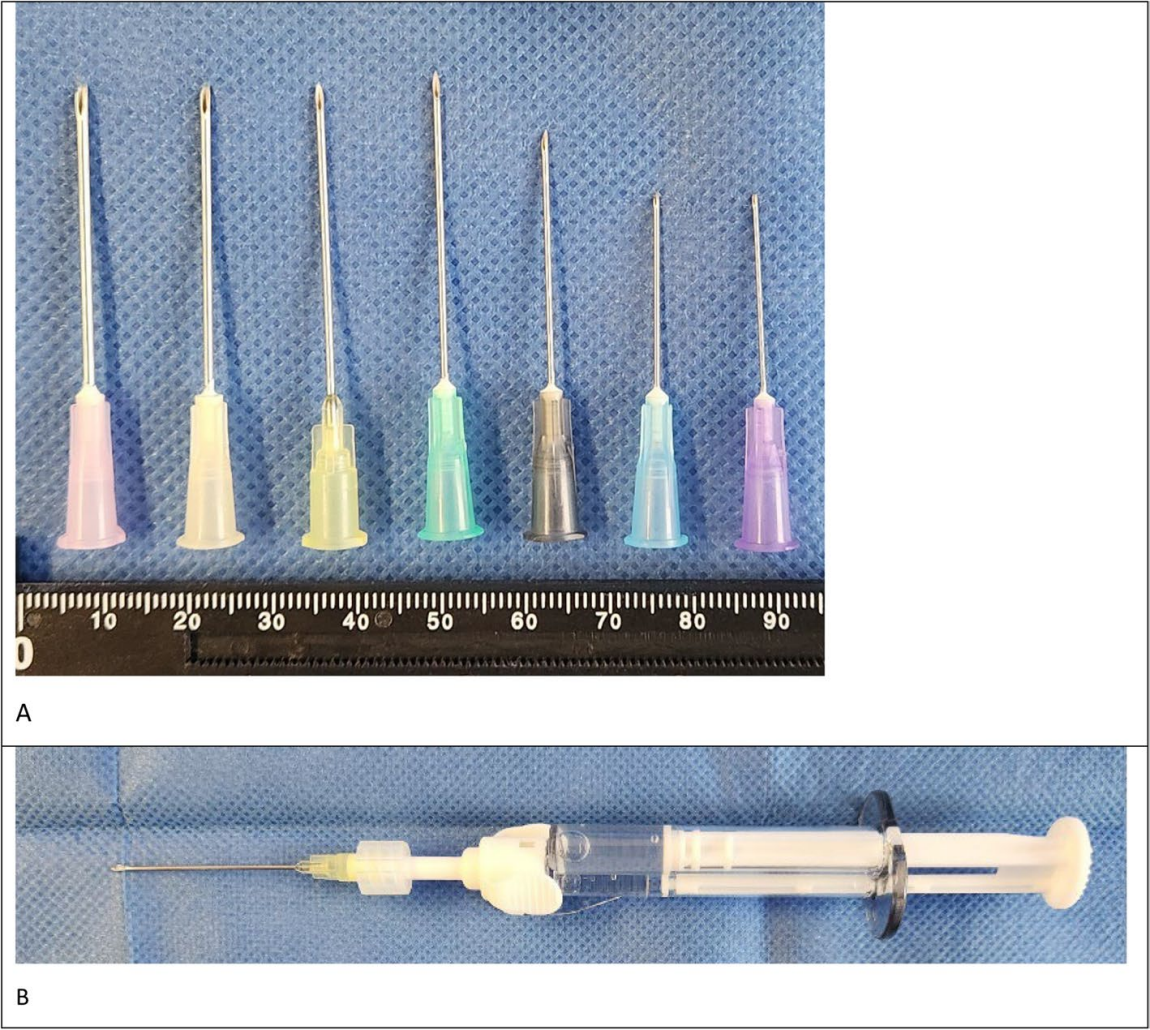
**A** Commercially available cannulas ranging from size G18 (1.20 × 40 mm) to G24 (0.55 × 25 mm). For arthroscopic injection, sizes G20 (0.90 × 40 mm) and G21 (0.8 × 40 mm) are the most suitable (yellow and green, respectively). **B** Injection-ready dual-chamber syringe (collagen type I and neutralization solution) with 20G cannula (Meidrix biomedicals GmbH, Esslingen, Germany)

For the application, a standard cannula was attached to the applicator of the Chondrofiller double syringe. The application of Chondrofiller was tested using several standard sizes of cannulas (Becton Dickinson GmbH, Heidelberg, Germany), ranging from G18 (1.20 × 40 mm) to G24 (0.55 × 25 mm). The handling of the cannula and the diameter size, ensuring smooth injection of the hydrogel, were carefully evaluated. It was found that a certain rigidity of the cannula, as well as a minimum length, is necessary for arthroscopically controlled injection. On the other hand, the diameter must allow for controlled and precisely targeted delivery into the defect. Cannulas of size G20 (0.90 × 40 mm) and G21 (0.8 × 40 mm) proved to be well-controllable while also enabling fine dosing (Fig. 2).

**Fig. 3:**
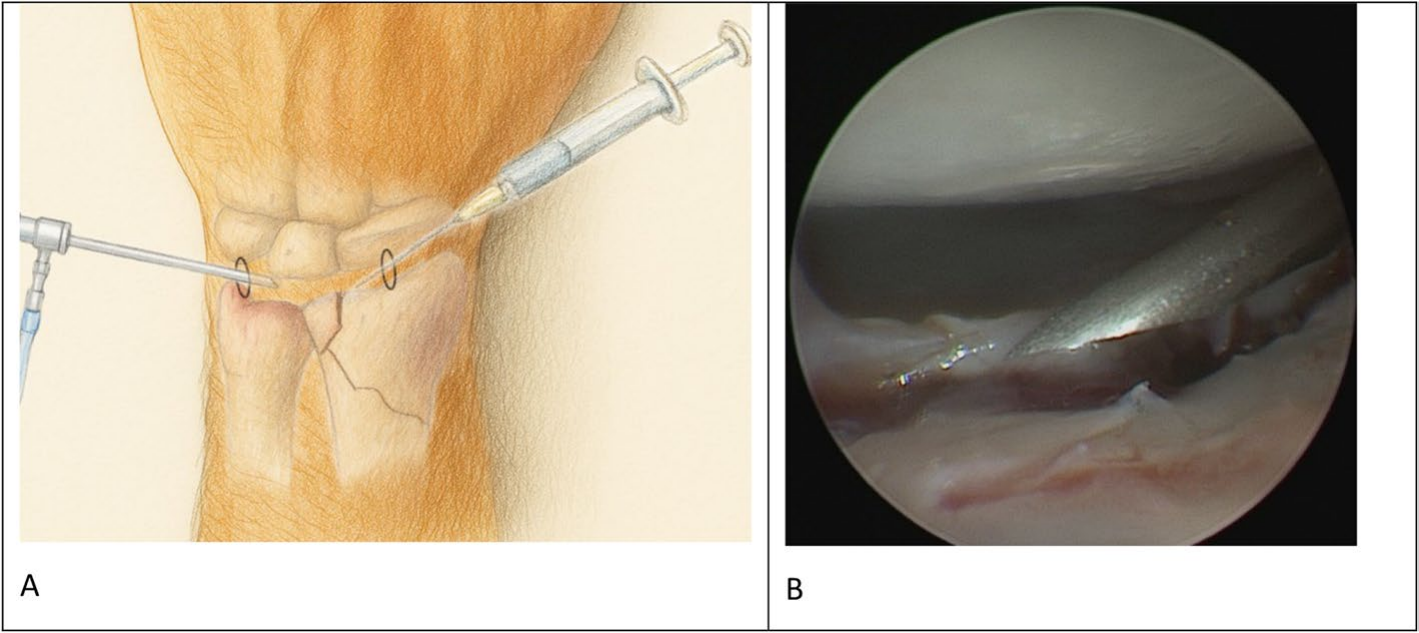
**A** Illustration of the arthroscopically controlled Chondrofiller application setup for treating persistent articular cartilage defects following ORIF of intra-articular radius fractures. Chondrofiller is inserted through a 20-G cannula via the 3/4 portal, with arthroscopic visualization via the 6R portal. (The illustration was created using ChatGPT, OpenAI, San Francisco, CA, USA). **B** In situ image of cartilage defect filling with Chondrofiller (Meidrix biomedicals GmbH, Esslingen, Germany) via 6R portal

After the intra-articular DRF has been reduced and treated with osteosynthesis under fluoroscopic control, wrist arthroscopy is performed to further inspect the joint surface. Gaps in the distal radius joint surface > 2 mm need to be re-reduced. Remaining gaps in the radius surface between 0.5 mm and ≤ 2 mm qualify for filling with Chondrofiller liquid. A dry arthroscopy is necessary to accurately apply the material into the defect. Any irrigation fluid must be removed by suction. The edges of the intra-articular gap should be debrided, and the gap itself should be cleared of debris using either a shaver or a probe. Once the gap is fully visible, the cannula can be inserted intra-articularly, and the defect should be filled flush under direct arthroscopic control. Depending on the defect’s location, the syringe can be inserted through the 6R portal to fill gaps in the fossa lunata and through the 3/4 portal to fill gaps in the fossa scaphoidea. The arthroscope is inserted through the respective free portal. For very radially located gaps, creating the 4/5 portal to insert the arthroscope may be helpful (Fig. 3).

After application, the Chondrofiller cures at body temperature. The sign of curing is the opacity of the hydrogel. Once this occurs, it is safe to stop arthroscopic traction. Postoperatively, a cast should be worn for approximately two weeks before initiating physiotherapy without weight bearing.

### Study results

Within the study period, a total of 59 intra-articular distal radius fractures (DRFs) were treated with arthroscopically assisted open reduction and internal fixation (ORIF) using volar locking plates. The cohort included 37 female patients (median age 62 years, range 27–85) and 22 male patients (median age 51 years, range 20–75). The most common fracture type was AO 23-C3 (*n* = 50, 84.7%), followed by 23-B2 (*n* = 5, 8.5%), 23-C2 (*n* = 3, 5.1%), and 23-B3 (*n* = 1, 1.7%) (Table 2).

**Table 2:** Number of intra-articular fracture types in the study population overall and in the subgroup treated with Chondrofiller for intra-articular gaps of 0.5 mm or ≤ 2 mm

AO Classification of Intra-Articular Fracture	Arthroscopically Assisted ORIF (all, *n* = 59)	Arthroscopically assisted ORIF Chondrofiller liquid applied (*n* = 25)
23-B 1	0	0
23-B 2	5 (8.5%)	2 (8%)
23-B 3	1 (1.7%)	1 (4%)
23-C 1	0	0
23-C 2	3 (5.1%)	0
23-C 3	50 (84.7%)	22 (88%)

In 25 of the 59 cases (42.4%), Chondrofiller liquid was applied intraoperatively to treat cartilage defects in the radial joint surface measuring between 0.5 and ≤ 2 mm. Defect width was assessed using intraoperative arthroscopic images (*n* = 20) or, when unavailable, based on postoperative radiographs according to the method by Lamijer et al. (*n* = 5) [46]. In cases of multiple chondral defects after fracture reduction, the largest was selected for analysis. The mean defect size was comparable between groups, with a mean of 1.4 mm (SD 0.5) in the Chondrofiller group and 1.3 mm (SD 0.7) in the non-Chondrofiller group (*P* = 0.36). All defects were full-thickness (Outerbridge and ICRS grade 4) with exposed subchondral bone.

Baseline characteristics did not differ significantly between treatment groups. In the Chondrofiller group (*n* = 25), 19 patients were female (76%, median age 62, range 33–85) and 6 males (24%, median age 50, range 20–64); in the non-Chondrofiller group (*n* = 34), 18 patients were female (53%, median age 62, range 27–84) and 16 males (47%, median age 52, range 23–75). Age was significantly higher in female than in male patients across the cohort (*P* = 0.015), but treatment allocation was not influenced by age (*P* = 0.88). A binomial test showed a trend toward female predominance (*P* = 0.067), but this was not statistically significant. There was also no significant difference in BMI between groups (Chondrofiller: mean 26.5 kg/m^2^, SD 6.4; no Chondrofiller: 23.8 kg/m^2^, SD 3.9; *P* = 0.054). Smoking status did not differ significantly (*P* = 0.801), though a significantly higher proportion of non-smokers was observed across the total cohort (77.6%; *P* = 0.001).

**Fig. 4:**
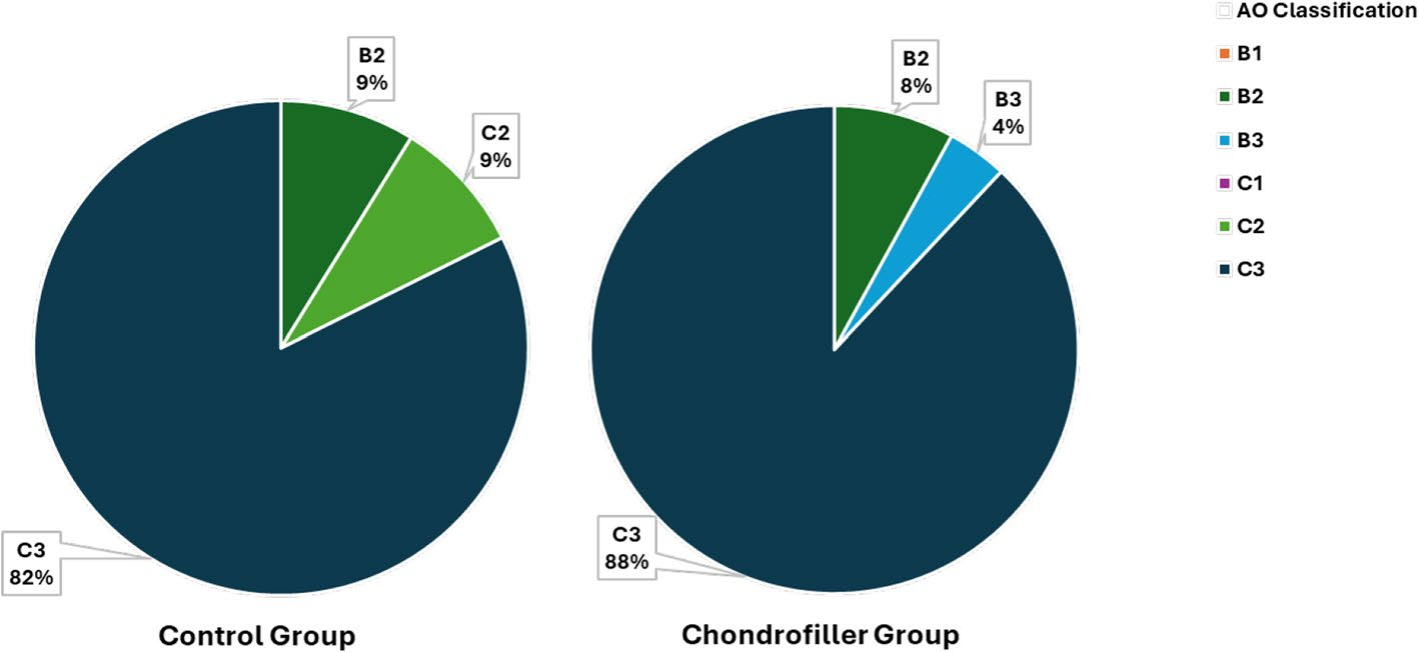
Distribution of AO Fracture Types in treatment and control groups: In both groups, type C3 fractures were predominant (88% in the Chondrofiller group and 82% in the control group)

In the subgroup treated with Chondrofiller, the most common fracture type was AO 23-C3 (*n* = 22; 88.0%), followed by B2 (*n* = 2; 8.0%) and B3 (*n* = 1; 4.0%). Among patients treated without Chondrofiller, 28 fractures were classified as AO 23-C3 (82.4%), 3 as B2 (8.8%), and 3 as C2 (8.8%). Overall, AO type 23-C3 accounted for 84.7% of all fractures treated (Fig. 4).

The distribution of AO fracture types did not differ significantly between the treatment groups (χ^2^(3) = 3.632, *P* = 0.3). No correlation could be demonstrated between AO classification and treatment modality (Spearman’s rho (*r* = 0.068, *P* = 0.611)). Due to the low frequency of non-C3 fractures, AO types were grouped into C3 vs. B2–C2. This dichotomized classification showed no significant association with Chondrofiller use, either (*P* = 0.7).

Time from fracture to surgery was similar in the group treated with Chondrofiller and the group not treated with Chondrofiller (mean 9 days, SD 6 vs. 8 days, SD 3). Operating time was slightly longer in the Chondrofiller group (104 min, SD 29) compared to the non-Chondrofiller group (92 min, SD 26). Side distribution was comparable, with right-hand injuries in 59.3% (non-Chondrofiller) vs. 45.8% (Chondrofiller) and left-hand injuries in 40.7% vs. 54.2%, respectively (*P* = 0.927). Chondral defect localization differed significantly between groups. Chondrofiller was applied significantly more often in isolated scaphoid fossa defects, while central defects affecting both fossae were more frequently observed in the non-Chondrofiller group (*P* = 0.03). Associated injuries were present in 51.5% of cases in the non-Chondrofiller group and 64.0% in the Chondrofiller group, with TFCC lesions being the most frequent in both. The distribution of concomitant injuries did not differ between groups (*P* = 0.28). At 6 weeks postoperatively, radiographic consolidation did not differ significantly between groups (*P* = 0.618), and no meaningful correlation was found (Spearman’s ρ = –0.085, *P* = 0.582), indicating that early bony healing was independent of Chondrofiller use.

The overall complication rate was low in both groups. In the non-Chondrofiller group, 2 of 14 patients (14.3%) experienced a marked range of motion limitation, while no cases of complex regional pain syndrome (CRPS) occurred. In the Chondrofiller group, 1 of 24 patients (4.2%) had postoperative mobility restriction, and CRPS was observed in 2 patients (8.3%). However, the distribution of complications showed no significant difference (χ^2^(2) = 2.317, *P* = 0.314), and no relevant correlation was detected (Spearman’s ρ = –0.008, *P* = 0.960).

### Follow-up arthroscopy in matched pairs: impact of Chondrofiller on cartilage regeneration

In 8 patients (3 females, 5 Males; median age 52 years, range 20–62) who received Chondrofiller to fill a cartilage defect in the radial joint surface during initial fracture fixation, follow-up arthroscopy was performed at the time of routine metal removal. The arthroscopies took place on average 10.4 months (SD 2.5) after the index procedure. The cartilage surface was evaluated according to the Outerbridge and ICRS classifications. Furthermore, the occurrence of fibrous tissue formation (FTF) was evaluated. The quantity of Chondrofiller applied during the initial surgery was documented intraoperatively as either flush or overfilled relative to the surrounding cartilage surface. These application levels were then correlated with the presence or absence of FTF observed during follow-up arthroscopy. The findings were compared to a control group of seven patients (5 Males, 2 females; median age 59 years, range 23–75) who had also sustained intra-articular distal radius fractures (DRF) and underwent ORIF combined with arthroscopy during initial treatment, but without Chondrofiller application. The re-arthroscopy of this matched control group (primarily matched by initial AO fracture type) was performed within the study period; however, the initial treatment was not necessarily conducted during the study period or at our clinic. The re-arthroscopy in this group was performed later, at an average of 13.2 months (SD 4.3) after the initial fracture treatment.

For the follow-up arthroscopy subgroup analysis, patients were stratified by fracture severity to allow for comparable evaluation across treatment groups. The Chondrofiller group (*n* = 8) included 2 B3 fractures (25.0%), 2 C2 fractures (25.0%), and 4 C3 fractures (50.0%). The comparison group without Chondrofiller (*n* = 7) comprised 2 B3 fractures (28.6%) and 5 C3 fractures (71.4%). This allowed for outcome assessment within similarly severe AO fracture types.

The size of any potential gap in the joint surface after initial ORIF was determined based on postoperative X-rays or CT images (Table 3) [46].

**Table 3:** Summary of fracture characteristics and follow-up findings in patients with or without Chondrofiller application during initial treatment. Patients treated with Chondrofiller showed significantly lower Outerbridge and ICRS grades (*P* = 0.03). FTF was observed predominantly in cases with overfilled Chondrofiller application, highlighting the importance of applying only the amount required to achieve a flush cartilage surface

	**Patient**	**Initial**			**Follow-OP**		
		**Fracture Type**	**Cartilage Gap (mm)**	**Filling quantity Chondrofiller**	**Outerbridge**	**ICRS**	**FTF**
Chondrofiller applied in the primary fracture treatment	Chon_1	C2	1.2	Flush	2	1	No
	Chon_2	C3	2.0	Overfilled	3	1	Yes
	Chon_3	B3	1.0	Flush	2	1	No
	Chon_4	C2	1.0	Overfilled	2	2	Yes
	Chon_5	C3	1.4	Overfilled	1	1	Yes
	Chon_6	B3	0.7	Overfilled	2	2	Yes
	Chon_7	C3	1.4	Overfilled	1	1	Yes
	Chon_8	C3	1.2	Flush	1	1	No
No Chondrofiller applied in the primary fracture treatment	NoChon_1	C3	1.1		4	3	
	NoChon_2	B3	0.9		4	2	
	NoChon_3	C3	0.7		3	3	
	NoChon_4	C3	0.9		3	3	
	NoChon_5	C3	1.5		2	2	
	NoChon_6	B3	1.7		3	3	
	NoChon_7	C3	2		3	3	

There was no statistically significant difference between groups in terms of age or sex distribution. In both groups, over 85% of patients were non-smokers, with no significant difference in smoking status. The side of the affected wrist was similarly distributed: 5 left and 3 right wrists in the Chondrofiller group versus 4 right and 3 left in the control group.

**Fig. 5:**
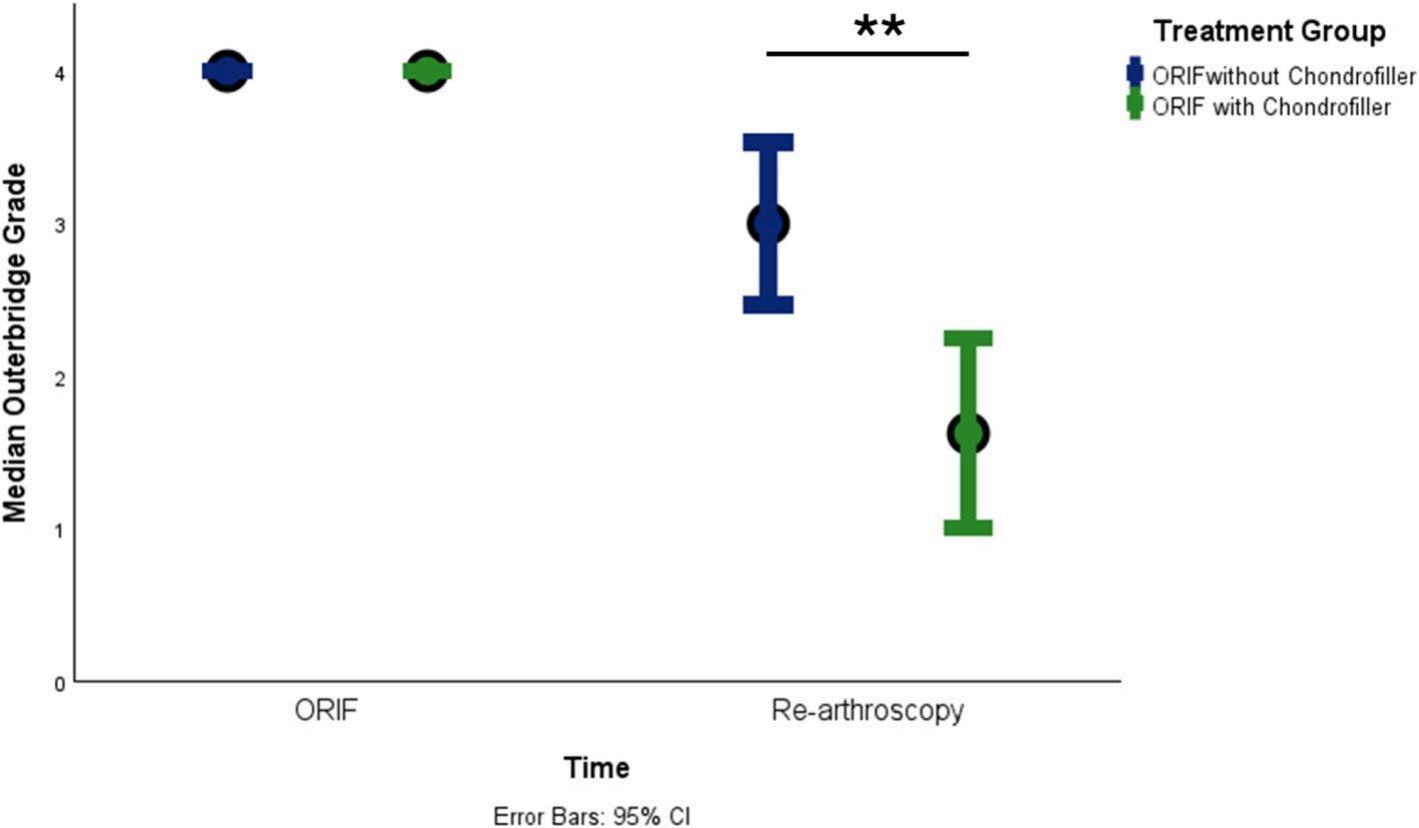
Median Outerbridge grade at ORIF and follow-up arthroscopy. Median Outerbridge grades (± 95% confidence intervals) are shown for the Chondrofiller group (green) and the control group (blue) at the time of ORIF and at follow-up re-arthroscopy

The compared groups did not differ significantly in fracture severity or in the width of the cartilage defects (Chondrofiller group: mean 1.2 mm, SD 0.4; no Chondrofiller: mean 1.3 mm, SD 0.5; *P* = 0.93). Regardless of initial fracture type and cartilage gap, the cartilage status at follow-up arthroscopy in the Chondrofiller group was favorable, with a median grade in the Outerbridge classification of 1.5 (range 1–3) and a median grade in the ICRS classification of 1 (range 1–2). In contrast, the control group without Chondrofiller showed less favorable outcomes, with a median grade in the Outerbridge classification of 3 (range 2–4) and a median grade in the ICRS classification grade of 3 (range 2–4). These differences were statistically significant (*P* = 0.006 for Outerbridge, *P* = 0.002 for ICRS) (Fig. 5).

**Fig. 6:**
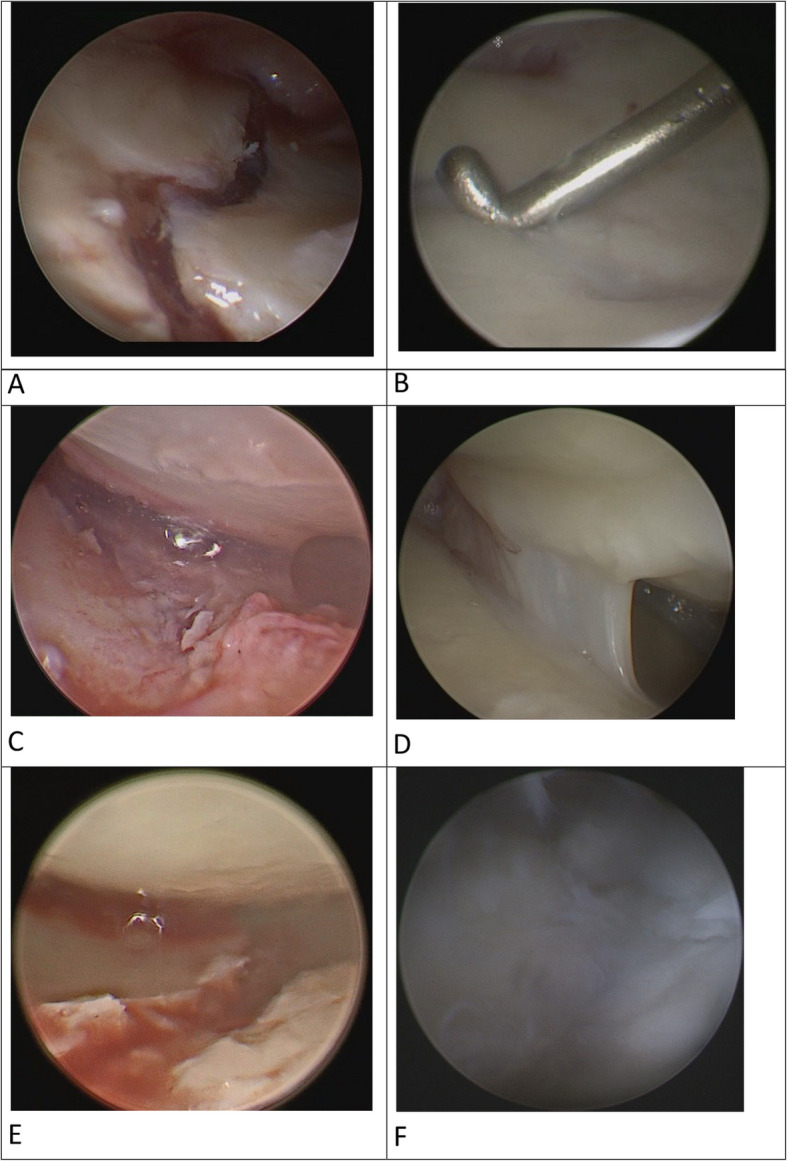
Comparison of intraoperative defect filling with Chondrofiller (**A**–**D**): (**A**) Flush filling of the defect (via 6R portal), (**B**) Follow-up result of the cartilage surface (via 3/4 portal), (**C**) Excessively filled intra-articular gap (via 3/4 portal), (**D**) Follow-up result: FTF (via 3/4 portal), and without use of Chondrofiller (**E**–**F**): (**E**) Native defect without use of Chondrofiller (via 3/4 portal), (**F**) Follow-up result: defect healing of the cartilage lesion (via 3/4 portal)

Fibrous tissue formations (FTFs) were observed in 5 out of 8 cases (62.5%) within the Chondrofiller group. Notably, all FTFs occurred exclusively in cases where the cartilage defect had been overfilled. In contrast, none of the three joints treated with a flush application of Chondrofiller showed FTF upon follow-up arthroscopy. These findings suggest that excessive filling may interfere with cartilage integration, while a precise, flush-level application appears to be associated with more favorable tissue response. (Fig. 6).

At six weeks, consolidation rates were comparable between groups (42.9% without vs. 37.5% with Chondrofiller). At final follow-up, one patient in the control group (14.3%) reported persistent pain and limited range of motion, whereas no complications were observed in the Chondrofiller group (0/8). Statistical analysis revealed no significant difference between groups in complication rates (*P* = 0.268).

## Discussion

Restoring joint congruence and preserving cartilage integrity are critical for preventing post-traumatic osteoarthritis (PTOA) following intra-articular distal radius fractures (DRFs). Step-offs and residual gaps ≥ 2 mm are significantly associated with the development of PTOA, which can lead to chronic pain, limited range of motion, and long-term functional impairment [16–21].

In this context, the quality and form of cartilage repair are essential for long-term outcomes, particularly when treating Grade 4 chondral lesions with exposed subchondral bone, as commonly observed in AO type B1–C3 intra-articular distal radius fractures.

Various cartilage repair techniques—such as autologous chondrocyte implantation, osteochondral autografts, and matrix-based approaches—have been extensively studied in large joints like the knee and hip [47–49]. However, these procedures often require multistage interventions and cell harvesting, which are not always practical or feasible in smaller joints such as the wrist and are therefore not a common treatment option.

The use of Chondrofiller, a cell-free type I collagen hydrogel, presents several practical advantages in the setting of wrist surgery. It enables single-stage application without the need for cell harvesting, while still supporting endogenous chondrocyte migration and proliferation. Its effectiveness in cartilage regeneration has been demonstrated in preclinical models and clinical studies in the knee and hip [38–40]. The present study is the first to investigate its technical feasibility and applicability in the wrist joint, where the small intra-articular space demands high precision and delicate instrumentation, as well as the correct technique.

In line with published recommendations, residual cartilage gaps larger than 2 mm should be reduced. Gaps below this threshold are generally considered acceptable in the literature. To address cartilage defects remaining after anatomical reduction, Chondrofiller was applied to residual cartilage gaps measuring between 0.5 and 2.0 mm [16–21]. The surgical technique described in this study—based on dry arthroscopy, careful debridement of defect margins, and the use of G20–G21 cannulas—provides a reproducible standard for safe and controlled application. For even smaller joints or defects, future developments may benefit from finer delivery systems.

A practical observation of this study was the mismatch between product volume and actual defect size. Although the smallest available 1 mL syringe was used, only about one-quarter of the hydrogel was needed to fill most defects, with the remainder discarded. This has economic and sustainability implications and suggests the need for smaller-volume packaging tailored to small joint indications.

The first follow-up arthroscopy results showed promising signs of improved cartilage regeneration in the Chondrofiller group. Patients treated with Chondrofiller demonstrated significantly lower Outerbridge and ICRS scores compared to the matched control group. These findings were independent of fracture type, initial defect size, age, or comorbidities, which were comparable between groups.

An important technical finding was the impact of filler volume on cartilage integration. All patients with a flush application of Chondrofiller remained free of fibrous tissue formation (FTF), whereas all overfilled cases developed FTF at follow-up. This suggests that excessive filler volume may impair tissue integration, likely due to mechanical irritation or disruption of the local biological environment. While FTF is a known occurrence after joint trauma [50], its selective appearance in overfilled cases highlights the importance of precise defect adaptation.

Interestingly, these findings contrast with experiences in larger joints such as the hip or femoral condyle, where slight overfilling is often tolerated. The wrist’s tight capsule, minimal synovial volume, and complex biomechanics appear to demand stricter volumetric control when using injectable scaffolds. The results of our study align with previous reports demonstrating the applicability of Chondrofiller Liquid in small joints. Corain et al. [51] evaluated its use in the trapeziometacarpal (CMC-1) joint for osteoarthritic cartilage lesions and reported positive outcomes in pain reduction, grip strength, and functional scores following a single fluoroscopically guided infiltration. Interestingly, the applied volume in the thumb joint ranged between 0.6 and 0.8 mL, carefully titrated according to patient feedback. In contrast, our intraoperative defect filling in the wrist joint required Markedly less volume, with only 0.2–0.3 mL used per case, highlighting the significantly smaller defect size after acute focal trauma compared to generalized degenerative conditions.

This study also provides valuable technical insights for future refinement of the procedure. The distribution of associated soft tissue injuries and consolidation rates did not differ significantly between groups, suggesting that cartilage treatment was the main determinant of chondral outcome.

The Outerbridge classification is a simple grading tool originally developed for retropatellar cartilage damage, but has since been established for use in other joints, including the wrist [52–56]. The ICRS classification is joint-independent. In cases of fracture-related cartilage damage, both classifications start at a grade 4 lesion. Follow-up assessments show similar results, although the ICRS grading tends to be more favorable. This difference may be due to the previously described inconsistent reliability of the Outerbridge classification [57, 58].

Limitations of this study include the small overall sample size, particularly in the follow-up arthroscopy subgroup, which limits statistical power and generalizability. The non-randomized design and treatment allocation based on intraoperative findings may have introduced selection bias, despite comparable baseline characteristics.

This study did not aim to evaluate postoperative outcomes. To correlate structural cartilage findings with clinically meaningful patient benefits, standardized functional measures, such as range of motion, grip strength, or validated patient-reported outcome measures (PROMs), should be further evaluated. Future studies should include larger, randomized cohorts with standardized functional assessments. Long-term follow-up is essential to determine whether Chondrofiller reduces the incidence of post-traumatic osteoarthritis and leads to lasting clinical improvement. Additionally, health-economic analyses are needed to assess whether its added cost is justified by potential long-term benefits, such as fewer reoperations, delayed osteoarthritis, and improved quality of life.

## Conclusion

This study demonstrates the technical feasibility and clinical applicability of Chondrofiller liquid for the treatment of post-traumatic cartilage defects in the wrist following intra-articular distal radius fractures. When applied to gaps between 0.5 and 2.0 mm, the cell-free collagen hydrogel showed promising short-term results, with improved cartilage quality observed during follow-up arthroscopy. Importantly, precise, flush-level application was critical to avoid fibrous tissue formation and ensure optimal integration. The procedure can be reliably performed arthroscopically using standard portals and fine cannulas, and offers a minimally invasive, single-stage treatment option without the need for cell harvesting. These findings provide a foundation for further clinical research and technical refinement in the application of biological scaffolds for cartilage regeneration in small joints. Larger studies with extended follow-up are warranted to confirm these early results and to evaluate the long-term functional and radiographic outcomes.

## Data Availability

No datasets were generated or analysed during the current study.
